# A Study on Real Time IGBT Junction Temperature Estimation Using the NTC and Calculation of Power Losses in the Automotive Inverter System

**DOI:** 10.3390/s21072454

**Published:** 2021-04-02

**Authors:** Heesun Lim, Jaeyeob Hwang, Soonho Kwon, Hyunjun Baek, Juneik Uhm, GeunHo Lee

**Affiliations:** Electric Motor Control Laboratory, Graduate School of Automotive Engineering, Kookmin University, Seoul 02707, Korea; popolhs@kookmin.ac.kr (H.L.); hjy910@kookmin.ac.kr (J.H.); qwer727@kookmin.ac.kr (S.K.); sexybaek91@kookmin.ac.kr (H.B.); fairwing@kookmin.ac.kr (J.U.)

**Keywords:** estimation of the junction temperature, IGBT, instantaneous power losses, inverter controller, thermal impedance model, real-time

## Abstract

This paper proposes a junction temperature estimation algorithm for the insulated gate bipolar transistor (IGBT) based on a power loss calculation and a thermal impedance model for inverter systems. The Simulink model was designed to calculate the power losses of power semiconductor devices and to estimate the junction temperature with a simplified thermal impedance model. This model can estimate the junction temperature up to the transient state, including the steady state. The parameters used to calculate the power losses, the thermal resistance, and the thermal capacitance were optimized for a given inverter to be tested for improving the accuracy. The simulation results and experimental measurement data were compared to verify the proposed junction temperature estimation algorithm. Finally, the algorithm was installed on the inverter controller, and the performance was verified by comparing the real time estimation result with the measured temperature.

## 1. Introduction

The temperature management strategy of an insulated gate bipolar transistor (IGBT), which is the power module of an inverter, has become an important issue with the increasing development of inverters for driving traction motors in electric vehicle application. Generally, current IGBTs are limited by the junction temperature, and the possibility of failure due to overheating tends to greatly increase unless the junction temperature is properly managed [1,2,3]. Recently, there have been numerous studies on methods for measuring the temperature of the IGBT indirectly by configuring the hardware to manage the junction temperature of the IGBT. One method measures temperature by attaching a negative temperature coefficient thermistor (NTC) near the junction. Most of the packaging modules provide a built-in NTC, but there is thermal resistance and thermal capacitance due to the physical separation distance, resulting in a difference from the junction temperature. Accordingly, correlation between the NTC temperature and the junction temperature is required [1,4]. Another method indirectly measures the junction temperature by monitoring the collector-emitter voltage during the on-state of the IGBT (i.e., the saturation voltage of IGBT). Another example is Mitsubishi’s J1 series. Companies such as semiconductor manufacturers provide a function to check the junction temperature by attaching a temperature sensor at a very close position to the junction [5]. In addition to the methods indirectly measuring the HW as described above, methods of estimating the junction temperature through thermal models have also been studied [6].

Previous studies are largely divided into analytical models [7,8,9], numerical models [10,11,12], and thermal network models [13,14,15,16,17,18] according to the method of designing the thermal model [19]. The thermal network models covered in this paper are divided into the Cauer and Foster thermal impedance models [20]. The Cauer thermal impedance model is based on the correlation between the IGBT module packaging structure and the physical property, and the Foster thermal impedance model is an equivalent model fitted from measurement results or simulation results. In the case of the thermal network model, valid results can be expected only within a limited range. It is difficult to consider temperature-dependent variables under conditions such as wide-range ambient temperature variations or transient conditions during operation at overloads or short-circuits [3]. Accordingly, many studies have been conducted to design an effective thermal impedance model. For example, there are studies about correcting the thermal impedance model considering temperature-dependent variables [3] or finite element method (FEM) thermal impedance models designed based on the physical structure of the IGBT module [3,21]. These studies improve the reliability of the estimated junction temperature. However, because most studies are simulation-based, the more complex the thermal impedance model, the more difficult it is to implement an algorithm that can estimate the junction temperature in the inverter in real time, although the complex thermal impedance model has higher accuracy. 

For this reason, in the field of mass-producing inverters for driving automobiles, to manage the junction temperature in real time when driving the inverter, the relationship between NTC and junction temperature is functionalized or tabled for various driving conditions to find the junction temperature. Due to the limitation of the memory of the inverter controller and the limitation of the time resource required to obtain experimental data, the area in which the junction temperature can be checked is limited to within a few hundred rpm compared to the operating range of the inverter for driving a vehicle from 12,000 to 20,000 rpm. In addition, even if the relationship between NTC and junction temperature is functionalized or tabled, it is difficult to respond to rapid changes in ambient temperature or changes in cooling conditions. This naturally leads to requiring a design margin according to the junction temperature when selecting the specifications of the IGBT module, which is directly connected to the increase in the inverter cost.

Therefore, this study focused on overcoming these limitations and designing a junction temperature estimation algorithm that can take into account variations in ambient and coolant temperatures, and can be used in inverter controllers. It also aimed at ensuring the junction temperature estimation performance over the entire operating range of the inverter. The algorithm designed for this should be as simple as possible while ensuring accuracy for various load patterns. First, the method of calculating the instantaneous power losses for each pulse width modulation (PWM) period is described in the current paper using the operating parameters of the IGBT provided in the datasheet and the variables sampled in real time by the inverter controller. Then, a simplified thermal impedance model is presented based on the NTC temperature of the IGBT module sampled by the inverter controller. The simulation results of the proposed algorithm are compared with the measured data to verify the reliability. Finally, the algorithms were implemented in the C language and applied to the inverter controller. The reliability of the estimation results was then verified by comparison with the measured temperature by performing under the load experiment on the dynamometer.

## 2. Calculation Power Losses of IGBT Modules

**Figure 1:**
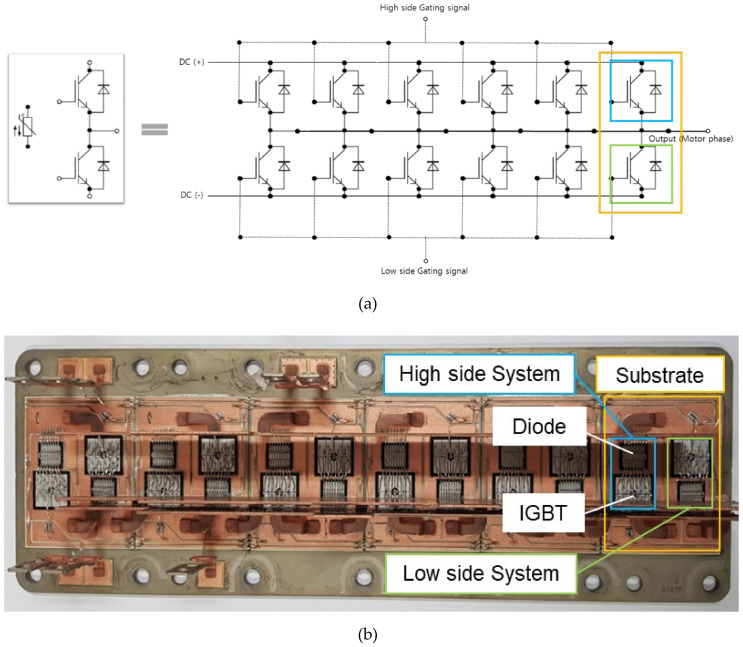
Structures of FF1000R17IE4. (**a**) Circuit diagram of FF1000R17IE4. (**b**) Six-parallel package structure of FF1000R17IE4.

The IGBT module used in this study is a FF1000R17IE4 shown in Figure 1, which provides a package for one phase. Specifically, a substrate with two IGBTs and two freewheeling diodes is arranged in six parallel structures. The power losses of the IGBT module can be explained by the sum of the IGBT power losses and the diode power losses:

(1) $$P_{total}=P_{IGBT}+P_{Diode}$$

The power losses of IGBT in Equation (1) can be divided into the switching losses occurring in the transient state (i.e., turn-on and turn-off state) and the conduction losses caused by the saturation voltage during the on-state of IGBT:

(2) $$P_{total[IGBT]}=P_{sw[IGBT]}+P_{cond\left[IGBT\right]}$$

(3) $$P_{cond\left[IGBT\right]}=V_{ce\left(sat\right)}\cdot I_{c}$$

(4) $$P_{sw\left[IGBT\right]}=\left(E_{on}+E_{off}\right)\cdot f_{sw}$$

Like the IGBT, the power losses of the diode can be divided into the conduction losses and the switching losses:

(5) $$P_{total[Diode]}=P_{sw[Diode]}+P_{cond[Diode]}$$

(6) $$P_{cond\left[Diode\right]}=V_{f}\cdot I_{f}$$

(7) $$P_{sw\left[Diode\right]}=E_{rec}\cdot f_{sw}$$

**Figure 2:**
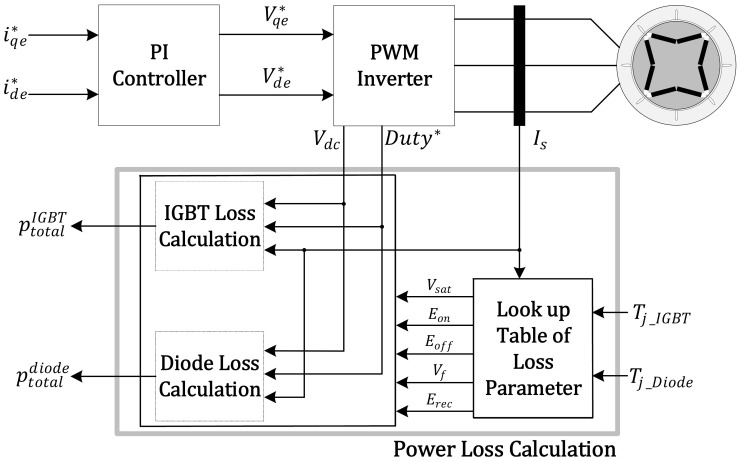
Block diagram of the power loss calculation algorithm.

In this study, the parameters related to the power losses on the IGBT datasheet were optimized for application. The method of optimizing the power loss parameters provided in the datasheet was applied to improve the accuracy of the power losses calculated in real time [22]. Optimized power loss parameters are provided as look-up tables, allowing parameters to be calculated in real time according to dependent variables (e.g., current, voltage, and junction temperature). The proposed method can calculate the power losses with the sampling variables (e.g., the terminal voltage, the phase current, the duty of PWM, and the switching frequency) according to the driving conditions and the optimized parameters of power losses. This proposed method was implemented as a Simulink model. The simulation results were compared with the experimental measurement data, and its validity is proved. A block diagram of the proposed method is shown in Figure 2.

### 2.1. Optimizing the Operating Parameters of IGBT

The collector-emitter saturation voltage of the IGBT depends on the collector-emitter voltages during the turn-off state of the IGBT, the collector current flowing through the IGBT, the junction temperature, and a given gate-emitter voltage. Semiconductor manufacturers already provide the saturation voltage ($V_{ce\left(sat\right)}$) characteristic curve in this regard. Therefore, based on this characteristic curve, the saturation voltage ($V_{ce\left(sat\right)}^{\prime }$) optimized for a given inverter to be tested was recalculated and applied as a look-up table. The Simulink model was constructed to calculate the saturation voltage ($V_{ce\left(sat\right)}^{\prime }$) according to the collector current flowing through the IGBT and junction temperature in real time. The saturation voltage ($V_{ce\left(sat\right)}^{\prime }$) optimized by recalculation using the saturation voltage ($V_{ce\left(sat\right)}$) characteristic curve can be expressed as follows:

(8) $$V_{ce\left(sat\right).Tj}^{\prime }\left(I\right)=V_{ce\left(sat\right).Tj}\cdot \frac{V_{ce\left(sat\right).Vge\_Inv}}{V_{ce\left(sat\right).Vge\_nom}}\cdot \frac{V_{dc.Inv}}{V_{dc.nom}}$$

(9) $$P_{cond\left[IGBT\right]}=V_{ce\left(sat\right).Tj}^{\prime }\left(I\right)\cdot I_{c}$$

**Figure 3:**
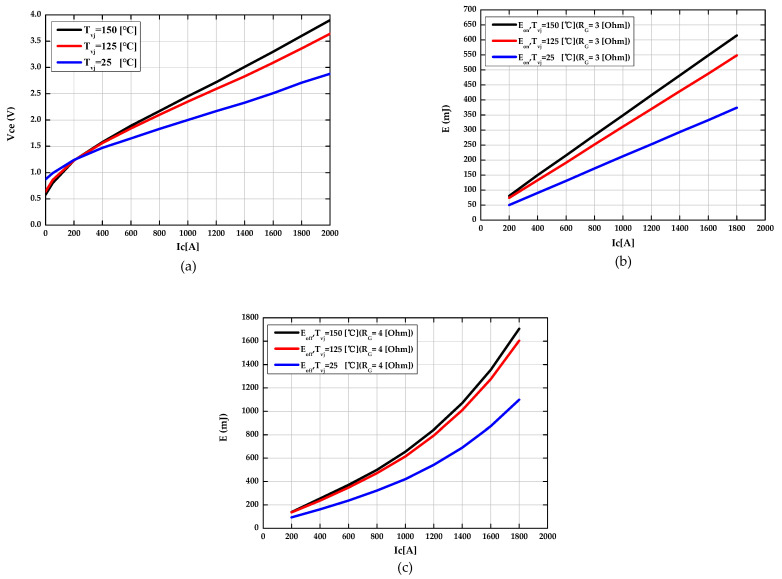
The result of optimization with power loss parameters of an insulated gate bipolar transistor (IGBT). (**a**) The optimized collector-emitter saturation voltage as a function of collector current at each different junction temperature. (**b**) The optimized turn-on switching losses as a function of collector current at each different junction temperature. (**c**) The optimized turn-off switching losses as a function of collector current at each different junction temperature.

Switching losses of the IGBT depend on the collector current flowing through the IGBT, the junction temperature, the given gate resistor, the gate-emitter voltages, and the collector-emitter voltages. The optimized switching losses ($E_{on}^{\prime },E_{off}^{\prime }$) were recalculated using the characteristic curve of switching losses ($E_{on},E_{off}$) provided in the datasheet. The Simulink model was then implemented using the look-up table to calculate the switching losses ($E_{on}^{\prime },E_{off}^{\prime }$) according to the collector current flowing through the IGBT and the junction temperature in real time. The recalculated switching losses ($E_{on}^{\prime },E_{off}^{\prime }$) are given by the following Equations (10) and (11). The switching losses of the IGBT are finally calculated as Equation (12) and the results are shown in Figure 3.

(10) $$E_{on.Tj}^{\prime }\left(I\right)=E_{on.Tj}\left(I\right)\cdot \frac{E_{on.Rg\_Inv}}{E_{on.Rg\_nom}}\cdot \frac{V_{dc.Inv}}{V_{dc.nom}}$$

(11) $$E_{off.Tj}^{\prime }\left(I\right)=E_{off.Tj}\left(I\right)\cdot \frac{E_{off.Rg\_Inv}}{E_{off.Rg\_nom}}\cdot \frac{V_{dc.Inv}}{V_{dc.nom}}$$

(12) $$P_{sw\left[IGBT\right]}=\left(E_{on}^{\prime }+E_{off}^{\prime }\right)\cdot f_{sw}$$

### 2.2. Optimizing the Operating Parameters of Diode

The conduction losses of the diode are caused by the voltage drop ($V_{f}$) during the on-state of the diode, which depends on the current flowing through the diode and the temperature of the junction. Therefore, the characteristic curve of the voltage drop ($V_{f}$) based on the current and the junction temperature is applied via the look-up table to calculate the voltage drop ($V_{f}^{\prime }$) according to the driving conditions. The voltage drop ($V_{f}^{\prime }$) is shown as Equation (13). The conduction losses of the diode are given by Equation (14).

(13) $$V_{f.Tj}^{\prime }\left(I\right)=V_{f.Tj}\left(I\right)$$

(14) $$P_{cond\left[Diode\right]}=V_{f.Tj}^{\prime }\left(I\right)\cdot I_{f}$$

**Figure 4:**
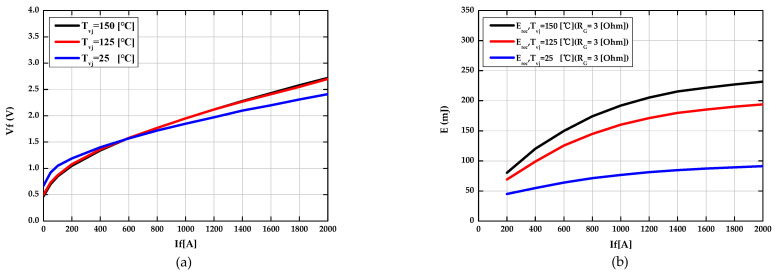
The result of optimization with power loss parameters of the diode. (**a**) The optimized forward voltage as a function of the forward current at each different junction temperature. (**b**) The optimized switching losses as a function of the forward current at each different junction temperature.

Switching losses of the diode are caused by reverse current in the reverse recovery area of the diode during turn-off. This is dependent on the value of the gate resistor for turn-on, the collector-emitter voltages during turn-off state, and the current flowing through the diode at the forward bias state. In addition, the switching losses energy ($E_{rec}^{\prime }$) optimized for the given inverter was recalculated by utilizing the characteristic curve of the switching losses energy ($E_{rec}$) provided in the datasheet and applied as the look-up table, so it can be calculated according to the current and the junction temperature in real time. The optimized switching losses ($E_{rec}^{\prime }$) is given by Equations (15) and (16) and the results are shown in Figure 4.

(15) $$E_{rec.Tj}^{\prime }\left(I\right)=E_{rec.Tj}\left(I\right)\cdot \frac{E_{rec.Rg\_Inv}}{E_{rec.Rg\_nom}}\cdot \frac{V_{dc.Inv}}{V_{dc.nom}}$$

(16) $$P_{sw\left[Diode\right]}=\left(E_{rec}^{\prime }\right)\cdot f_{sw}$$

### 2.3. The Simulation Result of IGBT Power Loss Calculation

To verify the algorithm that calculates the power losses in real time by applying the optimized power loss parameters as look-up tables, the inverter composed with the black paint module of IGBT was tested in the dynamometer environment. The data of power losses according to operating conditions were measured using a power analyzer (i.e., WT3000), and the simulation was performed by applying the same operating conditions. Considering the junction temperature over time to enhance the accuracy of simulation, the measured data with an IR camera were applied in the simulation. The power loss data obtained on the power analyzer were calculated using the root mean square (RMS) value as the current unit. The operating condition was thus selected as a stall condition to reduce errors due to the limitations of the measuring equipment. Other conditions were given randomly to verify the reliability of the power losses calculated within the wide operating range. The error of each experiment was calculated as the difference between the result of the simulation and the measured data. As a result, it was verified that the maximum error was 1.22%, which indicates high accuracy. Thus, it is possible to obtain the power losses for estimating the junction temperature using the optimized power loss parameters look-up tables and variables (e.g., the phase current, the PWM duty, and the DC-link voltage) of the inverter controller. The simulation results for each experiment are shown in Table 1.

## 3. Design of the Simplified Thermal Impedance Model Using NTC

**Figure 5:**
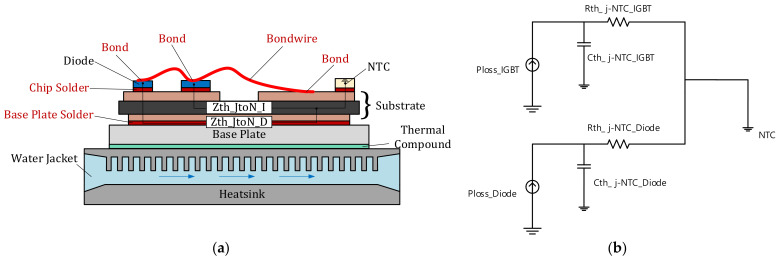
The proposed thermal impedance model from the junction to the NTC. (**a**) The proposed thermal circuit model shown in a schematic cross section of an IGBT module including the heatsink and water jacket. (**b**) The proposed thermal equivalent circuit diagram.

The described research results are based on the experimental method to design the Foster model. The thermal impedance model proposed in this paper is the thermal impedance model that has an NTC, which is packaged in an IGBT module to provide the temperature of the baseplate as the ground node, rather than a model that has the coolant temperature (or ambient temperature) as the ground node. In the case of the existing thermal circuit model, it is common to select a node based on a vertical structure ranging from coolant to thermal grease, baseplate, solder, substrate, solder, and junction. This can, however, differ largely from the actual thermal impedance because the factors to be considered in the design process are complex and it is not easy to obtain accurate data. In addition, it is difficult to consider the effect of the change in temperature, and flow rate of the cooling water and the thermal grease. In the case of the NTC, it is affected by the coolant temperature (or ambient temperature) to the same degree as the junction of the IGBT module. This means that this model has an advantage of being able to react indirectly to a changing coolant temperature (or ambient temperature). When the NTC is equalized to the junction with one thermal impedance, the effect of cooling water and thermal grease is affected by both the NTC and the junction, such that the influence can be indirectly reflected by the temperature change of the NTC. In this case, even if there is a difference in the influence of the junction and the NTC on the cooling water and the thermal grease, it can be assumed that the error in the thermal impedance caused by the difference is very small compared to the thermal impedance due to the separation distance between the NTC and the junction. In addition, because both the NTC and the junction are located on the substrate, the temperature data can be easily measured. To implement the C language-based algorithm, the thermal impedance model was designed with one thermal resistance and one thermal capacitance by simplifying it as much as possible. The proposed thermal impedance model is shown in Figure 5. Figure 5a is the proposed circuit model shown with a schematic cross section of an IGBT module. A heatsink with a water jacket is a commonly used cooling structure in the traction inverter. The baseplate is attached on the heatsink with thermal compound. The substrate is soldered onto the baseplate. The IGBT and the diode is soldered onto the substrate and the NTC is also soldered. The packaged circuit is connected with bond wire. The thermal impedance between the NTC and the junction are expressed as just one thermal impedance value. Figure 5b shows the simplified thermal circuit diagram. The ground node sets with temperature of the NTC.

The experimental data were obtained by measuring the NTC temperature and the junction temperature under actual load conditions according to the reference method for the Foster model [17] to calculate the thermal resistance and the thermal capacitance of the proposed thermal impedance model. The temperature data of the two measuring points are applied to Equation (17), which was used to obtain the thermal impedance over time. Finally, the thermal impedance model of the junction and the NTC can be obtained by extracting the thermal resistance and the thermal capacitance through curve fitting with the thermal impedance obtained over time. The form of curve fitting is equal to Equation (18).

(17) $$Z_{th\_jN}\left(t\right)=\frac{\Delta T_{jN}\left(t\right)}{P_{loss}}=\frac{T_{junction}\left(t\right)-T_{NTC}\left(t\right)}{P_{loss}}$$

(18) $$Z_{th\_jN}\left(t\right)=R_{th\_jN}(1-e^{-\frac{t}{R_{th\_jN}\cdot C_{th\_jN}}})$$

### 3.1. The Thermal Impedance Model between Junction and NTC

**Figure 6:**
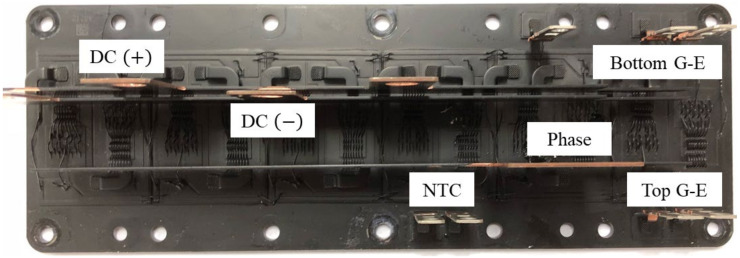
Black paint module of the FF1000R17IE4.

**Figure 7:**
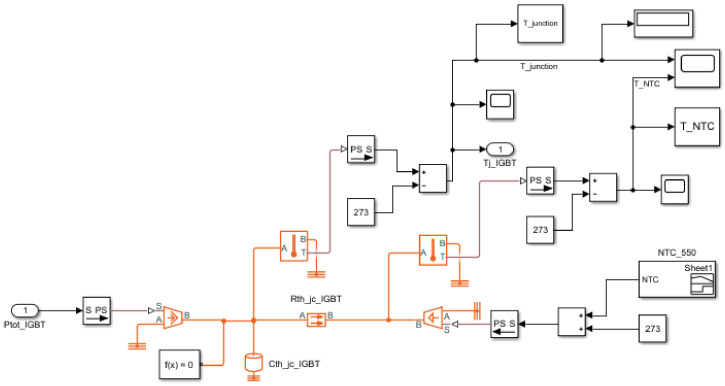
Simulink model of the proposed the thermal impedance model.

To design the thermal impedance model from the junction to the NTC, the temperature of the NTC and the junction temperature were acquired in real time under the load test conditions in a dynamometer environment. The temperature of the NTC was measured with a temperature measuring instrument with a sampling time of 10 ms. The temperature of the junction was measured with an IR camera using the black paint module shown in Figure 6. To obtain an accurate thermal impedance, it is first important to measure each temperature accurately. The purpose of this study is to design the simplest thermal impedance model to secure junction temperature estimation performance, so the process of designing thermal impedance is very important. To ensure the junction temperature estimation performance in all operating areas, various experimental cases were selected. The load test conditions were selected within the operating range of the inverter required by the system. Each experimental condition was selected according to the DC-link voltage (*V*), the switching frequency (*kHz*), the coolant temperature (°C), load (%), and fundamental frequency (*Hz*). The FF1000R17IE4 used in this experiment has a 6-parallel structure. As shown in Figure 7, six high-side IGBTs and six low-side diodes are arranged on the upper side, and six low-side IGBTs and six high-side diodes are arranged on the lower side. Therefore, the thermal impedance model for stall condition and driving condition were independently extracted because the thermal impedance has a difference according to whether the fundamental frequency in the operating condition exists. The remainder of the experiment conditions were randomly selected and conducted within the operating range of the inverter. A total of 40 experiments were conducted according to each condition to obtain experimental data. The thermal impedance graph was obtained using the temperature data measured for each experimental condition through Equation (17). The thermal resistance and thermal capacitance were then extracted by fitting the thermal impedance graph. It was confirmed that the standard deviation of the calculated thermal resistance and thermal capacitance for all experimental cases was 1.16 for the constraint condition and 0.17 for the driving condition. Therefore, the final thermal resistance and thermal capacitance were selected as average values. The results in Table 2 were selected as average values. In the stall condition, the average thermal resistance of the IGBT was 41.13 (°C/*kW*) and the average thermal resistance of the diode was 102.1 (°C/*kW*). The average thermal capacitance of the IGBT was 11.21 (*W*/°C) and the average thermal capacitance of the diode was 3.36 (*W*/°C). This is because the area of the IGBT is bigger than the area of the diode. In the driving condition, the average thermal resistance of the IGBT was 54.76 (°C/*kW*) and the average thermal resistance of the diode was 155.59 (°C/*kW*). The average thermal capacitance of the IGBT was 13.56 (*W*/°C) and the average thermal capacitance of the diode was 5.16 (*W*/°C). The reason why the result of the driving condition is larger than that of the stall condition is due to the switching pattern along the current path. In the stall condition, the inverter’s switching state is fixed for DC current and heat is generated in six IGBTs and six diodes. However, in the driving condition, the inverter’s switching state changes for AC current and heat is generated in 12 IGBTs and 12 diodes. 

### 3.2. The Simulation Result of Junction Temperature Estimation

Simulation was performed by implementing the thermal impedance model. The thermal resistance and the thermal capacitance obtained through the experiment were applied in Simulink. The temperature of the NTC was applied as raw data experimentally acquired for each operating condition, and the power losses were calculated from the power loss calculation model designed in Section 2 and applied as the input. There is a disadvantage in that the error present in the power loss calculation model is reflected as the error of the estimated junction temperature, but simulation verification was performed in consideration of this to confirm the reliability of the junction temperature estimation algorithm during the final implementation stage. The thermal impedance model implemented in Simulink is shown in Figure 7, and the simulation results for the operating conditions are shown in Table 3. The error of the estimated temperature for each case was calculated based on the maximum temperature over time, and the maximum error was 1.5% for the IGBT and 1.3% for the diode. The high accuracy of these results indicates that the designed thermal impedance value was close to the actual impedance value and also proved that even a simple thermal impedance model with the NTC as a ground node can obtain sufficient estimation performance.

## 4. Experimental Verification of the Junction Temperature Estimation Algorithm

The power loss calculation algorithm verified through simulation and the thermal impedance model based on the NTC must be implemented as C language-based software to mount the inverter controller. When designing software, an operation period must be selected in consideration of the execution time, and an error occurs when there is a decrease in resolution according to the operation period. In addition, an error that is not confirmed in the simulation may additionally occur due to the potential error of the sampling variables used in the calculation. Therefore, it is necessary to verify whether the software is properly designed by comparing the result of the junction temperature estimation performed by the micro controller unit (MCU) and the actual temperature data through an experiment. In the experimental case, to check the temperature estimation performance for the overall driving range of the inverter, the entire driving speed range including the stall condition was considered, and the load pattern was also selected in various ways.

### 4.1. Experimental Setup

**Figure 8:**
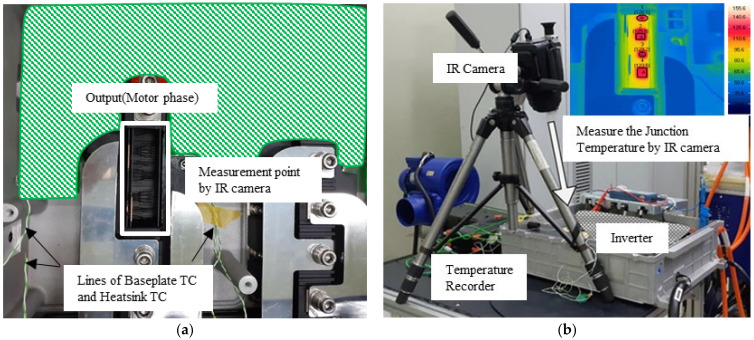
Experimental setup to verify the proposed algorithm. (**a**) Inverter with black paint module assembled. (**b**) Inverter installed in a dynamo environment, IR camera, and temperature recorder.

A black paint module was set up on the inverter shown in Figure 8. The junction temperature was measured by the IR camera. The data of the inverter (the estimated junction temperature, the calculated power losses, and the temperature of the NTC) were obtained by the controller area network (CAN) Analyzer with a sampling time of 100 ms.

The designed software consists of two functions: a power loss calculation algorithm and a thermal impedance model-based junction temperature estimation algorithm. The power loss parameter of the power loss calculation algorithm was applied as a lookup table and calculated through interpolation according to the driving conditions. At this time, the execution time could not be converged within the PWM period due to interpolation, so the operation period was set to 2 ms. Because the operation time was longer than the PWM period, the resolution was lowered, which in turn caused an error in the power loss calculation result. Finally, it was reflected as an error of the estimated temperature. To reduce the error due to the software limitation, the integral value of the power losses per 2 ms were applied in the stall condition because the integral value of the power losses due to the instantaneous current was the same as the integral value of the power losses due to the average current of 2 ms. By comparison, in the driving condition, as the frequency increases, an error increases between the integral value of the power losses due to the instantaneous current and the integral value of the power losses due to the average current of 2 ms. To solve this problem, the software was designed to predict the instantaneous power loss value by multiplying the sine wave of the fundamental frequency by the maximum power loss value.

**Figure 9:**
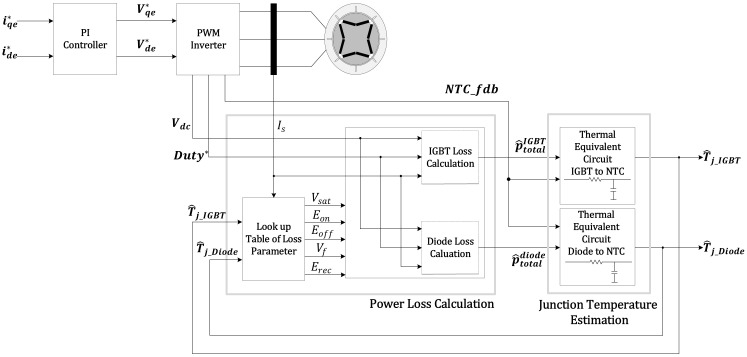
Block diagram of the proposed junction temperature estimation algorithm.

Experimental conditions were selected with the sequence of torque command, the switching frequency, the fundamental frequency, the temperature of coolant, and the DC-link voltage. The block diagram of the proposed junction estimation algorithm is shown in Figure 9. The algorithm has two main functions. One is the thermal impedance model consisting of the thermal resistance and the thermal capacitance from the junction to the NTC. This function needs the results of the power loss calculation function, with each IGBT and diode as the inputs. The other is the power loss calculation function. This function needs the inputs that have the information for power loss parameters (e.g., the phase current, the duty of the PWM, the temperature of the NTC, the switching frequency, and the feedback, as a result of the estimated junction temperature). Finally, the results of this function will be the power losses of the IGBT and diode. These results will be fed back to the function of the thermal impedance model.

### 4.2. Experimental Results

The experimental results were classified according to the presence or absence of the fundamental frequency and analyzed by dividing them into the stall condition and the driving condition.

#### 4.2.1. The Results of the Experiment at the Stall Condition

Table 4 shows the experiment results. For each experimental case, the error between the estimated value and the measured value of each case was calculated based on the maximum temperature over all data. As a result, the error was up to 2%. A value of 2% means that the maximum error is very similar to that of the simulation result, which was 1.3% for the IGBT and 1.5% for the diode. To mount the designed algorithm on the inverter controller, the software must be designed in consideration of the problem that the operation cycle cannot be shortened due to the execution time problem.

**Figure 10:**
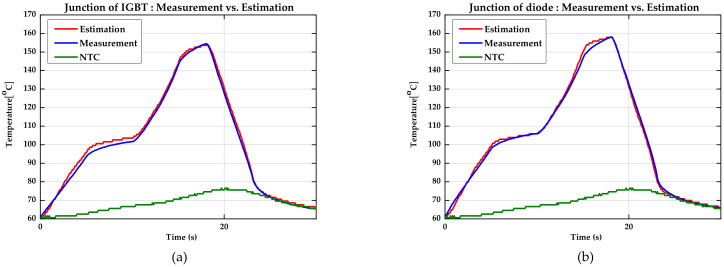
Comparison of estimation results in the stall condition (Case 1). (**a**) The estimated junction temperature of the IGBT (red), the measured junction temperature of the IGBT (blue), and the measured temperature of the NTC (green). (**b**) The estimated junction temperature of the diode (red), the measured junction temperature of the diode (blue), and the measured temperature of the NTC (green).

In addition, when considering the potential errors of the sampled variables, an error of 2% proves that the software is designed properly. Figure 10 shows graphs with the result of the experiment (i.e., the estimated temperature, the measured temperature, and the temperature feedback from the NTC) for Case 1. 

With this result, it can be known that the slope at which the temperature of the NTC rises in the transient period during which the current rises is lower than the junction temperature. The junction temperature has a very steep rising slope. This means that it takes time for heat to be conducted due to the physical separation distance from the IGBT and diode chip, which are the heating points, to the NTC. In addition, the temperature of the junction can be two times the temperature of the NTC. This makes it difficult to manage the junction temperature only by observing the temperature of the NTC.

By comparison, the junction temperature estimated by the proposed algorithm converges with very little error in the entire section compared to the actual temperature. Furthermore, the estimated junction temperature converges to the actual temperature even in the state where the current does not flow and the temperature drops to the steady state. After cooling sufficiently, the junction temperature converges close to the temperature of the NTC. The reason why the temperature of the NTC is slower than the cooling rate seems to be the difference due to the shape of the channel, where the coolant flows directly under the baseplate where the IGBT and diode chip are located. The fact that the junction temperature converges to the temperature of the NTC when no current flows means that it is reasonable to assume that the junction temperature and the temperature of the NTC are affected equally by the ambient and the coolant temperature because the junction and NTC are on the same layer. Therefore, by designing the thermal impedance model based on the NTC, the external factors affecting the junction temperature can be considered indirectly. 

#### 4.2.2. The Results of the Experiment at the Driving Condition

**Figure 11:**
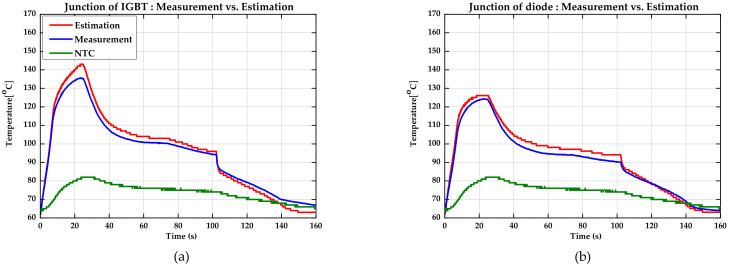
Comparison of estimation results in the driving condition (Case 1). (**a**) The estimated junction temperature of the IGBT (red), the measured junction temperature of the IGBT (blue), and the measured temperature of the NTC (green). (**b**) The estimated junction temperature of the diode (red), the measured junction temperature of the diode (blue), and the measured temperature of the NTC (green).

The specifications of this traction motor-inverter system are listed in Table 5. Table 6 shows the experiment results in the driving condition. As in the stall condition, the error for each experiment was calculated based on the maximum temperature. The maximum error was 5.46%. For Cases 1, 2, and 3, the junction temperature was estimated while driving along the speed-torque (S-T) curve of the interior permanent magnet synchronous motor (IPMSM). The S-T curve ranged from 100 to 12,000 rpm based on the maximum output torque required by the system. Other test cases had a fixed speed. Through these experiments, it was possible to verify the performance of the proposed algorithm while operating with the maximum load applied up to the maximum fundamental frequency required for the inverter in the automotive traction system. The estimation results for Case 1 are shown in Figure 11. The results of estimating the junction temperature while operating along the S-T curve show a larger error than the result when the rpm is fixed at a low speed. Looking at Figure 11, it can be seen that a certain error continues to exist in the region where the fundamental frequency is high. The errors that occurred mostly fell within 3%. In addition to the transient region in which the junction temperature rises rapidly, the result of tracking the behavior of the actual junction temperature in the entire region can be confirmed. When the proposed algorithm is applied, it is advantageous in that the thermal behavior of the junction temperature can be estimated with high accuracy in the inverter controller, even when the required operating range is wide, as in an automotive traction system.

## 5. Conclusions

The power loss calculation algorithm of this paper calculates the instantaneous power losses for each switching period, which were used as an input to the thermal impedance model. Therefore, the junction temperature can be estimated in real time and in the steady state or the transient state using the proposed algorithm. In addition, the thermal impedance model of the NTC to the junction has meaningful results in estimating the junction temperature. These results were verified experimentally by comparing the estimated and measured temperatures. The error of the constraint condition was within 2%, and the error of the driving condition was confirmed to be within 6%. This is proof that the method of designing the power loss calculation algorithm and the temperature estimation algorithm using the NTC-based thermal circuit model is reasonable. In particular, it showed that it is possible to achieve not only a similar performance to the generally thermal impedance model, but also practicality. Above all, the simplified thermal circuit model based on the NTC calculated each thermal impedance for various load patterns during the design process, and the thermal impedance was selected as an average value taking into account the deviation of each case. As a result, it was shown that the temperature estimation performance can be secured by showing a narrow error distribution, with only the initial design values of the thermal circuit model without a separate correction process. Therefore, it can be applied to mass production sufficiently if additional verification is performed in consideration of the error distribution that may occur when applying the temperature estimation algorithm to mass-produced products. In addition, compared to conventional methods, the design margin due to junction temperature can be reduced, which is expected to reduce inverter costs. On this point, the proposed algorithm of this paper would be useful in estimating the junction temperature of a water-cooled inverter for automotive systems in real time, and preventing the failure of power modules by overly high temperatures.

## Figures and Tables

**Table 1:** The simulation results of the power loss calculation model.

Condition of Experiment						Results of Power Loss Calculation				
Case	Coolant (°C)	*f_sw_* (*kHz*)	*f_e_* (*Hz*)	$V_{dc}$ $\left(V\right)$	$T_{ref}$ $\left(\%\right)$	Measurement	Simulation			Error
						Total $\left(Kw\right)$	Total $\left(Kw\right)$	IGBT $\left(Kw\right)$	Diode $\left(Kw\right)$	$\left(\%\right)$
1	65	4	0	550	66	1.62	1.626	1.14	0.486	0.38
2	65	8	0	550	30	1.64	1.628	1.18	0.448	0.73
3	65	4	0	680	58	1.53	1.539	1.098	0.441	0.59
4	65	4	0	810	50	1.729	1.747	1.261	0.486	1.04
5	25	4	0	550	100	2.43	2.439	1.718	0.721	0.37

**Table 2:** $R_{th}$ and $C_{th}$ from the junction to the NTC extracted by experiments in the stall condition and the driving condition.

Result	IGBT		Diode	
	$R_{thjN\_IGBT}$ (°C/*kW*)	$C_{thjN\_IGBT}$ (*W*/°C)	$R_{thjN\_Diode}$ (°C/*kW*)	$C_{thjN\_Diode}$ (*W*/°C)
Stall condition	41.13	11.21	102.1	3.36
Driving condition	54.76	13.56	155.59	5.16

**Table 3:** The simulation results of the junction temperature estimation with the proposed thermal impedance model in various conditions of the inverter.

Condition of Inverter Driving						Measurement vs. Simulation Result					
Case	*Coolant* (°C)	$f_{sw}$ (*kHz*)	$f_{e}$ (*Hz*)	$V_{dc}$ (*V*)	$T_{ref}$ (%)	IGBT			Diode		
						$T_{measure}$ (°C)	$T_{est}$ (°C)	*Error* (%)	$T_{measure}$ (°C)	$T_{est}$ (°C)	*Error* (%)
1	65	4	0	550	100	153.8	158.9	1.1	155.5	158.7	0.25
2	65	4	0	550	100	153	157.2	1.5	155.3	158.5	0.8
3	65	4	0	550	100	155.4	160.2	1.3	157.4	160.7	0.3
4	65	4	0	550	100	157	161.8	1.2	158.9	162.2	0.25
5	65	4	0	550	100	155	159.1	1.0	156.6	159.9	0.5
6	65	8	33.3	550	100	138.2	135.3	0.94	139.5	136.7	1.03
7	65	8	33.3	680	100	146.1	139.4	0.54	146.9	141	1.14
8	65	8	33.3	810	100	146.1	136.4	0.75	147.2	138.2	1.3

**Table 4:** The experiment results of each case for verification of the proposed junction estimation algorithm in the stall condition.

Condition of Experiment Case						Measurement vs. Estimation					
Case	*Coolant* (°C)	$f_{sw}$ (*kHz*)	$f_{e}$ (*Hz*)	$V_{dc}$ (*V*)	$T_{ref}$ (%)	IGBT			Diode		
						$T_{measure}$ (°C)	$T_{est}$ (°C)	*Error* (%)	$T_{measure}$ (°C)	$T_{est}$ (°C)	*Error* (%)
1	65	4	0	550	100	153.7	152.3	0.91	157	156.7	0.19
2	65	4	0	680	80	144.9	146.3	0.97	145.5	145.6	0.07
3	65	4	0	710	80	149.5	150.1	0.4	149.2	148.4	0.54
4	65	4	0	810	60	134.2	137	2.0	132.9	132.3	0.45
5	65	4	0	550	100	154.4	153.6	0.52	158.1	157.9	0.13
6	65	4	0	550	100	154.5	152.4	1.36	157.9	156.8	0.7

**Table 5:** The specifications of the traction motor-inverter system.

Specifications		Value
DC-link voltage	Minimum	550 (V)
	Nominal	710 (V)
	Maximum	810 (V)
Motor speed	Range	0 to 12,000 (rpm)
Torque	Maximum	500 (Nm)
Power	Maximum	120 (kW)
Phase current	Maximum	636 (A)
Switching frequency	0 to 500 (rpm)	4 (kHz)
	500 to 12,000 (rpm)	8 (kHz)

**Table 6:** The experimental results of each case for verification about the proposed junction estimation algorithm in the driving condition.

Condition of Experiment Case						Experiment vs. Estimation					
Case	*Coolant* (°C)	$f_{sw}$ (*kHz*)	$f_{e}$ (*Hz*)	$V_{dc}$ (*V*)	$T_{ref}$ (%)	IGBT			Diode		
						$T_{measure}$ (°C)	$T_{est}$ (°C)	*Error* (%)	$T_{measure}$ (°C)	$T_{est}$ (°C)	*Error* (%)
1	65	8	TN	550	100	135.6	143	5.46	124.3	126.1	1.45
2	65	8	TN	680	80	134	138.5	3.36	124.6	125.6	0.8
3	65	8	TN	810	80	151.5	155.6	2.71	144.3	146. 6	1.59
4	65	8	33.3	550	80	109.9	113.2	3.0	109.2	109.5	0.27
5	65	8	33.3	680	80	122.9	124.9	1.63	114.6	118	2.97
6	65	8	20.0	710	80	95	94.9	0.72	95.8	97.9	2.19
7	65	8	33.3	710	80	126.7	127.5	0.63	117.5	119.4	1.62
8	65	8	33.3	710	80	144.2	147.4	0.84	130.9	133.4	3.16
9	65	8	33.3	810	80	139.5	138.5	2.22	126.5	126.5	1.91

## Data Availability

Data sharing is not applicable to this article.

## List of Symbols

$C_{th}$	the thermal capacitance
$C_{th\_jN}$	the thermal capacitance between junction and NTC
$E_{on}$	the energy of turn-on losses per switching cycle
$E_{off}$	the energy of turn-off losses per switching cycle
$E_{rec}$	the energy of reverse-recovery losses per switching cycle
$f_{e}$	the frequency of electric angle
$f_{sw}$	the switching frequency
$i_{de}^{*}$	the command of d-axis current
$I_{c}$	the collector current
$I_{f}$	the forward current
$i_{qe}^{*}$	the command of q-axis current
$P_{cond[Diode]}$	the conduction losses of the diode
$P_{cond\left[IGBT\right]}$	the conduction losses of the IGBT
$P_{Diode}$	the power losses of the diode
$P_{IGBT}$	the power losses of the IGBT
$P_{sw[Diode]}$	the switching losses of the diode
$P_{sw[IGBT]}$	the switching losses of the IGBT
$P_{total}$	the total power losses of the IGBT module
$P_{total[Diode]}$	the total power losses of the diode
$P_{total[IGBT]}$	the total power losses of the IGBT
$R_{th}$	the thermal resistance
$R_{th\_jN}$	the thermal resistance between junction and NTC
$T_{est}$	the temperature of estimation
$T_{junction}$	the temperature of junction
$T_{measure}$	the temperature of measurement by IR camera
$T_{NTC}$	the temperature of NTC
$T_{ref}$	the torque reference
$V_{ce\left(sat\right)}$	the collector-emitter saturation voltage
$V_{dc}$	the DC-link voltage
$V_{de}^{*}$	the voltage command of d-axis current controller
$V_{f}$	the forward voltage
$V_{qe}^{*}$	the voltage command of q-axis current controller
$Z_{th\_jN}$	the thermal impedance between junction and NTC
**Indices**	
c	collector
cond	conduction
de	d-axis of synchronously rotating reference frame
e	electric angle
est	estimation
f	forward bias
fdb	feedback
Inv	inverter
j	junction
jN	junction to NTC
nom	nominal
qe	q-axis of synchronously rotating reference frame
rec	reverse-recovery
ref	reference
Rg	gate resistor
sat	saturation
sw	switching
th	thermal
Vge	gate-emitter voltage

## References

[1] Infineon Technologies (2009). Using the NTC inside a Power Electronic Module.

[2] Hu Z., Ge X., Xie D., Zhang Y., Yao B., Dai J., Yang F. (2019). An Aging-Degree Evaluation Method for IGBT Bond Wire with Online Multivariate Monitoring. Energies.

[3] Wu R., Wang H., Ma K., Chimire P., Iannuzzo F., Blaabjerg F. A temperature-dependent thermal model of IGBT modules suitable for circuit-level simulations. Proceedings of the IEEE Energy Conversion Congress and Exposition.

[4] Martin S., Ma X. Correlating NTC-Reading and Chip-Temperature in Power Electronic Modules. Proceedings of the PCIM Europe 2015, International Exhibition and Conference for Power Electronics, Intelligent Motion, Renewable Energy and Energy Management.

[5] Seiichiro I., Mikio I. (2015). J-Series IPM and T-PM for EV and HEV Applications.

[6] Kim Y.S., Sul S.K. On-Line Estimation of IGBT Junction Temperature Using On-State Voltage Drop. Proceedings of the Conference Record of 1998 IEEE Industry Applications Conference. Thirty-Third IAS Annual Meeting.

[7] Mantooth H.A., Hefner A.R. (1997). Electro-thermal simulation of an IGBT PWM inverter. IEEE Trans. Power Electron..

[8] Ishiko M., Kondo T., Usui M., Tadano H. A compact calculation method for dynamic electro-thermal behavior of IGBTs in PWM inverters. Proceedings of the 2007 Power Conversion Conference-Nagoya.

[9] Du B., Hudgins J.L., Santi E., Bryant A.T., Palmer P.R., Mantooth H.A. (2010). Transient electrothermal simulation of power semiconductor devices. IEEE Trans. Power Electron..

[10] Lakhsasi A., Hamri Y., Skorek A. (2001). Partially coupled electro-thermal analysis for accurate prediction of switching devices. Proc. Can. Conf. Electr. Comput. Eng..

[11] Riccio M., Irace A., Breglio G., Spirito P., Napoli E., Mizuno Y. Electro-thermal instability in multi-cellular trench-IGBTs in avalanche condition: Experiments and simulations. Proceedings of the 2011 IEEE 23rd International Symposium on Power Semiconductor Devices and ICs (ISPSD).

[12] Greco G., Vinci G., Bazzano G., Raciti A., Cristaldi D. Layered electro-thermal model of high-end integrated power electronics modules with IGBTs. Proceedings of the IECON 2014-40th Annual Conference of the IEEE Industrial Electronics Society (IECON).

[13] Hefner R., Blackburn D.L. (1993). Simulating the dynamic electrothermal behavior of power electronic circuits and systems. IEEE Trans. Power Electron..

[14] Yun C.S., Malberti P., Ciappa M., Fichtner W. (2001). Thermal component model for electrothermal analysis of IGBT module systems. IEEE Trans. Adv. Packag..

[15] Kojima T., Yamada Y., Nishibe Y., Torii K. Novel RC compact thermal model of HV inverter module for electro-thermal coupling simulation. Proceedings of the 2007 Power Conversion Conference-Nagoya (PCC).

[16] Castellazzi A., Ciappa M., Fichtner W., Batista E., Dienot J., Mermet-Guyennet M. Electro-thermal model of a high-voltage IGBT module for realistic simulation of power converters. Proceedings of the ESSDERC 2007-37th European Solid State Device Research Conference.

[17] Gragger J.V., Fenz C.J., Kernstock H., Kral C. A fast inverter model for electro-thermal simulation. Proceedings of the 2012 Twenty-Seventh Annual IEEE Applied Power Electronics Conference and Exposition (APEC).

[18] Batard C., Ginot N., Antonios J. (2015). Lumped dynamic electrothermal model of IGBT module of inverters. IEEE Trans. Compon. Packag. Manuf. Technol..

[19] Qian C., Fan J., Tang H., Sun B., Ye H., Zhang G. (2018). Thermal management on IGBT power electronic devices and modules. IEEE Access.

[20] Infineon Technologies (1995). Transient Thermal Measurements and Thermal Equivalent Circuit Models.

[21] Arash N., Ashkan N., Osama A.M. A Physics-Based, Dynamic Electro-Thermal Model of Silicon Carbide Power IGBT Devices. Proceedings of the 2013 Twenty-Eighth Annual IEEE Applied Power Electronics Conference and Exposition (APEC).

[22] Infineon Technologies (1999). Calculation of Major IGBT Operating Parameters.

